# Catastrophic Floods May Pave the Way for Increased Genetic Diversity in Endemic Artesian Spring Snail Populations

**DOI:** 10.1371/journal.pone.0028645

**Published:** 2011-12-20

**Authors:** Jessica Worthington Wilmer, Lynde Murray, Ché Elkin, Chris Wilcox, Darren Niejalke, Hugh Possingham

**Affiliations:** 1 Biodiversity and Geosciences Program, Queensland Museum, Brisbane, Queensland, Australia; 2 School of Biological Sciences, University of Queensland, Brisbane, Queensland, Australia; 3 Marine and Atmospheric Research Division, Commonwealth Scientific and Industrial Research Organisation (CSIRO), Hobart, Tasmania, Australia; 4 Olympic Dam Expansion Project, BHP Billiton, Adelaide, South Australia, Australia; Biodiversity Insitute of Ontario - University of Guelph, Canada

## Abstract

The role of disturbance in the promotion of biological heterogeneity is widely recognised and occurs at a variety of ecological and evolutionary scales. However, within species, the impact of disturbances that decimate populations are neither predicted nor known to result in conditions that promote genetic diversity. Directly examining the population genetic consequences of catastrophic disturbances however, is rarely possible, as it requires both longitudinal genetic data sets and serendipitous timing. Our long-term study of the endemic aquatic invertebrates of the artesian spring ecosystem of arid central Australia has presented such an opportunity. Here we show a catastrophic flood event, which caused a near total population crash in an aquatic snail species (*Fonscochlea accepta*) endemic to this ecosystem, may have led to enhanced levels of within species genetic diversity. Analyses of individuals sampled and genotyped from the same springs sampled both pre (1988–1990) and post (1995, 2002–2006) a devastating flood event in 1992, revealed significantly higher allelic richness, reduced temporal population structuring and greater effective population sizes in nearly all post flood populations. Our results suggest that the response of individual species to disturbance and severe population bottlenecks is likely to be highly idiosyncratic and may depend on both their ecology (whether they are resilient or resistant to disturbance) and the stability of the environmental conditions (i.e. frequency and intensity of disturbances) in which they have evolved.

## Introduction

Despite the immediate and seemingly far reaching devastation that follows intense natural disturbances, there is an extensive literature showing that such events can ultimately generate environmental conditions favourable to the survival, growth and diversification of living organisms and ecosystems, at least as measured by species diversity [1]–[7]. Recently, studies have shown parallel responses to disturbance between species and genetic diversity with variation between-sites (beta diversity) increasing and within-sites (alpha diversity) decreasing [8]–[10]. However, within species the impact of catastrophes which result in severe reductions in population sizes are neither predicted nor known to result in the promotion of genetic diversity. Rather, the outcome of such population bottlenecks is usually the loss of genetic variation, the severity of which depends on both the intensity and duration of the crash and the subsequent rate of recovery and immigration [11]–[16]. Furthermore, while genetic diversity has been shown to enhance species and even ecosystem resilience to catastrophic disturbances [17]–[19], the reverse i.e. catastrophes enhancing genetic diversity, is yet to be demonstrated.

Circumstances allowing for testing the impact of catastrophes on any aspect of ecosystem and evolutionary dynamics are highly unusual due to the longitudinal nature of the work required combined with serendipitous timing. Some notable exceptions are studies of droughts and Darwin's finches in the Galapagos [20], hurricanes and spider and lizard communities on Caribbean islands [21]–[23] and cyclones and silvereye populations on Heron Is in the Great Barrier Reef [24]. However, having the complementary longitudinal genetic datasets to directly test population genetic consequences of natural disasters is even more exceptional.

Our study of the artesian spring ecosystem of the Great Artesian Basin (GAB) in arid central Australia has provided an unparalleled opportunity to examine the impact of periodic, catastrophic disturbances on the genetic diversity of the endemic fauna that inhabit the springs. Australia's GAB is one of the largest artesian systems in the world, covering 22% of the continent [25] and is of national cultural, economic and biological significance. Artesian springs form when geological structures allow water from the GAB to reach the surface, forming freshwater spring pools in an otherwise inhospitable desert environment [25]. This is a naturally patchy and fragmented ecosystem with the springs likened to “aquatic islands in an arid sea” [26] and they support a wealth of endemic species including plants, arachnids, crustaceans, molluscs and fish [27]–[30].

Arid central Australia is subject to recurrent but irregular significant floods due to unpredictable heavy rainfall events. Native vegetation is highly responsive to such rainfall variations and the rivers, creeks and terminal drainage lakes, which are intermittent to ephemeral, are transformed by floodwaters into vast inland seas often extending thousands of square kilometres [31]. Such events are the focus of well-documented and spectacular “boom” and “bust” cycles in many animal populations [32]–[34]. However, for the endemic fauna of the artesian springs the impact of such flood events is hitherto unknown.

Here we investigate for the first time the effect of a catastrophic flood event on the artesian spring ecosystem of arid central Australia. Long term monitoring of the springs in our study area has allowed us to directly test the population genetic consequences of the flood by examining how genetic diversity and divergence estimates and effective population sizes of one of the endemic spring invertebrates (the hydrobiid snail, *Fonscochlea accepta*), changed through time.

## Materials and Methods

### Artesian springs and our study site

An archetypal artesian spring is comprised of three parts: a shallow vent or pool where artesian water first reaches the surface, an outflow tail of shallow water and wetland vegetation which grows around the pool and tail and where the substrate allows successive layers of carbonate. Springs can range from simple damp mounds and surface seeps to large deep pools with rapidly flowing drainages that support extensive wetland areas. Springs can be no higher than a few centimetres above ground level or rise up to over 10 m in height and up to 30 m in diameter. Whether or not springs form mounds depends on a variety of factors including water discharge rates, the sediment composition and the nature and concentration of dissolved mineral salts; wind blown debris and plant materials can also contribute to mound formation [26]. Since the springs are produced by faults or where the aquifer outcrops at the surface the springs tend to be spatially clustered. Due to this clustering, springs have previously been arranged along a fine to broad scale spatial hierarchy as individual springs, spring groups, spring complexes and then spring supergroups [25]. This hierarchy is derived solely from an assessment of the spatial patterns of the physical location of the springs, and is not based on empirical measurement of connectivity among springs for organisms.

**Figure 1:**
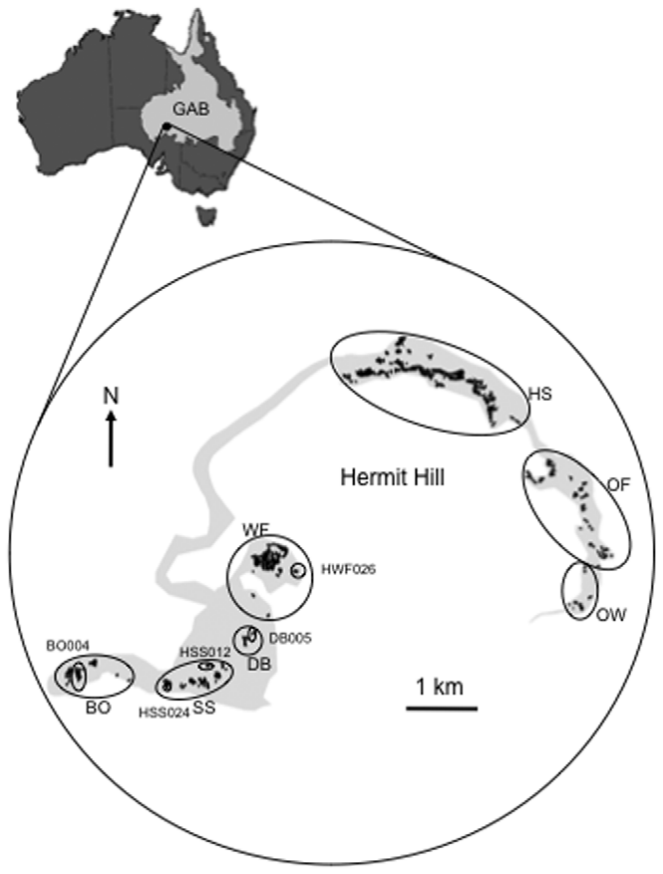
Location of the Hermit Hill spring complex within the Australian Great Artesian Basin (GAB). The complex is comprised of seven previously identified spring groups (Bopeechee - BO, Dead Boy - DB, Hermit Hills - HS, Old Finniss - OF, Old Woman - OW, Sulphuric - SS and West Finniss - WF). Our five study springs are located in four of these springs groups: BO004 (Bopeechee), DB005 (Dead Boy), SS012 and SS024 (Sulphuric) and WF026 (West Finniss). The large flood channel, which demarcates the separate drainage basins (NE drainage – HS/OF/OW; SW drainage – BO/SS/DB and West Finniss) within the Hermit Hill complex is shown on the map in grey.

**Figure 2:**
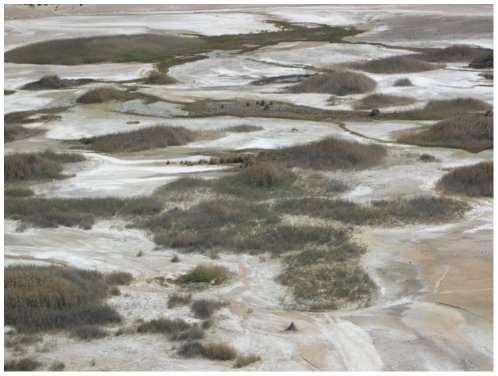
Artesian springs of the West Finniss spring group. Aerial photo depicting springs typical of the groups that comprise the Hermit Hill spring complex. The West Finniss group is comprised of both active (flowing ground water) and inactive (dry) springs that lie at ground level and do not form prominent raised mounds. The natural patchiness and inhospitable nature of the surrounding arid habitat of the artesian springs ecosystem is also clearly shown. Photo credit: Drew Tyre.

Our study focuses on the Hermit Hill spring complex in northern South Australia (29° 32′ 34″ S, 137° 26′ 29″ E). This spring complex is part of the Lake Eyre Supergroup [25] situated on the south-west margin of the GAB (Fig. 1). The Hermit Hill spring complex contains seven previously identified spring groups (Fig. 1), with each group being comprised of between 6 and 263 individual springs. The spring groups lie around the base of Hermit Hill (relative elevation of Hermit Hill = 123 m) and occur in two spatially separated drainage basins; three spring groups (Hermit Hill Springs (HS), Old Finniss (OF), Old Woman (OW)) are situated in the drainage basin to the north east of Hermit Hill and another three groups (Bopeechee (BO), Sulphuric (SS), Dead Boy (DB)) are in a basin south west of Hermit Hill (Fig. 1). While West Finniss (WF) is spatially associated with the southwest drainage it is considered an isolated system i.e. separated from any other group by its drainage patterns. The individual springs of the Hermit Hill complex do not form large conspicuous mounds, lying at or just above ground level (see Fig. 2).

### Ethics statement

No specific permits were required for the described field studies. Notification and appreciation of access to the study site was provided to the tradition owners of the GAB springs country especially Reg Dodd and the Aboriginal Lands Trust. The community of native species dependent on natural discharge of water from the Great Artesian Basin is listed as “endangered” under Australia's Environment Protection and Biodiversity Conservation (EPBC) Act 1999.

### Long term ecological monitoring and temporal samples

Local draw-down of artesian water can result in the extinction of local springs. Major concerns for the maintenance of the springs in the study area arose during the development of the Olympic Dam mine project (BHP Billiton formerly Western Mining Corporation) because of the potential for draw-down effects from the mine's water supply borefields and their proximity to the Hermit Hill spring complex [35], [36]. These concerns prompted the establishment of major hydrogeological, botanical and zoological monitoring programs on a set of individual springs, which have been ongoing since baseline studies were carried out in 1983. In the Hermit Hill complex, 16 springs from the seven previously defined spring groups were chosen as monitoring springs.

In conjunction with these monitoring programs, recent large scale population genetic and ecological studies have focused intensively on a subset of four of the seven spring groups incorporating 5 of the 16 monitoring springs (Bopeechee (HBO004), Dead Boy (HDB005), Sulphuric (HSS012 and HSS024) and West Finniss (HWF026)) [37]–[39]. These studies have allowed temporally continuous sampling from 2002–2006. In addition to these years, historical samples from the long term monitoring programs were available from these 5 springs for the years 1988–1990 and 1995, though not all springs had samples for each of those years.

### Study species and sampling

The springs in our study area support at least six species of hydrobiid snails in two genera (*Trochidrobia* and *Fonscochlea*). We have focused our analysis on the species *Fonscochlea accepta* as it is both widespread and abundant in the springs at our study site, its life cycle and natural history are relatively well described and is found in both the spring vents and outflows [27].

Springs are sampled in late winter/early spring (August/September), when temperatures in the Australian arid interior are cooler and evaporative water loss from the springs is lowest. In order to obtain a representative sample of snails from each spring we collected sediment samples along a transect passing from the spring vent to the bottom of the main outflow tail. The number of samples taken was proportional to spring size with a minimum of ten taken in each spring. Sediment samples for each individual spring were aggregated together (eliminating any differences that might be due to microspatial variation within a spring) then preserved in the field using ethanol. Samples were returned to the lab where they were sorted and *F. accepta* identified using a dissecting microscope. Where contemporary and historical sampling allowed, 25 snails per spring per year were genotyped resulting in an overall total of approximately 900 genotyped individuals from the 5 monitoring springs (Table 1).

**Table 1:** Temporal sample sizes, genetic diversity estimates and inbreeding coefficients.

Spring ID/Year Sampled	No. Snails	No of Loci	Mean Allelic Richness	Mean Heterozygosity		F_IS_
				Observed	Expected	
Bopeechee 004						
1988	25	5	2.6	0.279	0.255	−0.118
1989	25	5	2.5	0.218	0.195	−0.124
1990	25	5	2.2	0.235	0.182	**−0.305**
2002	25	5	3.3	0.232	0.216	−0.077
2003	25	5	3.1	0.231	0.211	−0.098
2004	25	5	3.0	0.192	0.210	0.089
2005	20	5	3.1	0.235	0.236	0.002
2006	25	5	3.0	0.180	0.200	0.100
Dead Boy 005						
1988	25	5	2.5	0.222	0.231	0.043
1989	26	5	2.7	0.202	0.201	−0.004
1990	25	5	2.3	0.336	0.254	**−0.332**
1995	11	5	2.3	0.253	0.213	−0.197
2002	25	5	2.4	0.164	0.185	0.118
2003	25	5	2.7	0.200	0.200	0.002
2004	25	5	2.3	0.176	0.185	0.047
2005	25	5	2.5	0.216	0.199	−0.089
2006	25	5	2.5	0.208	0.221	0.062
Sulphuric 012						
1988/1989*	15	5	2.4	0.284	0.219	−0.311
1995	13	5	3.5	0.274	0.272	−0.008
2002	25	5	3.5	0.252	0.271	0.072
2003	25	5	3.7	0.342	0.296	−0.161
2004	25	5	3.3	0.293	0.271	−0.080
2005	25	5	3.3	0.264	0.261	−0.010
2006	25	5	3.5	0.260	0.289	0.102
Sulphuric 024						
1988	18	5	2.5	0.244	0.216	−0.134
1989	20	5	2.7	0.310	0.244	−0.281
1990	20	5	2.4	0.305	0.214	**−0.446**
2002	25	5	3.0	0.220	0.234	0.063
2003	25	5	3.1	0.272	0.240	−0.136
2004	25	5	3.2	0.244	0.220	−0.112
2005	25	5	3.1	0.204	0.209	0.023
2006	25	5	2.9	0.220	0.204	−0.078
West Finniss 026						
1988	25	5	2.9	0.207	0.207	0.010
1989	22	5	2.5	0.307	0.258	−0.281
1990	9	5	1.8	0.306	0.245	**−0.612**
2002	33	5	3.3	0.222	0.236	0.063
2003	25	5	3.3	0.232	0.241	0.039
2005	25	5	3.2	0.252	0.227	−0.111
2006	20	5	2.9	0.190	0.206	0.077

*There were <5 individual snails available for SS012 from 1989 and were therefore combined with the 1988 samples.

F_IS_ values in bold are significant at the p<0.05 level.

### Severe flood event and its ecological impact

**Figure 3:**
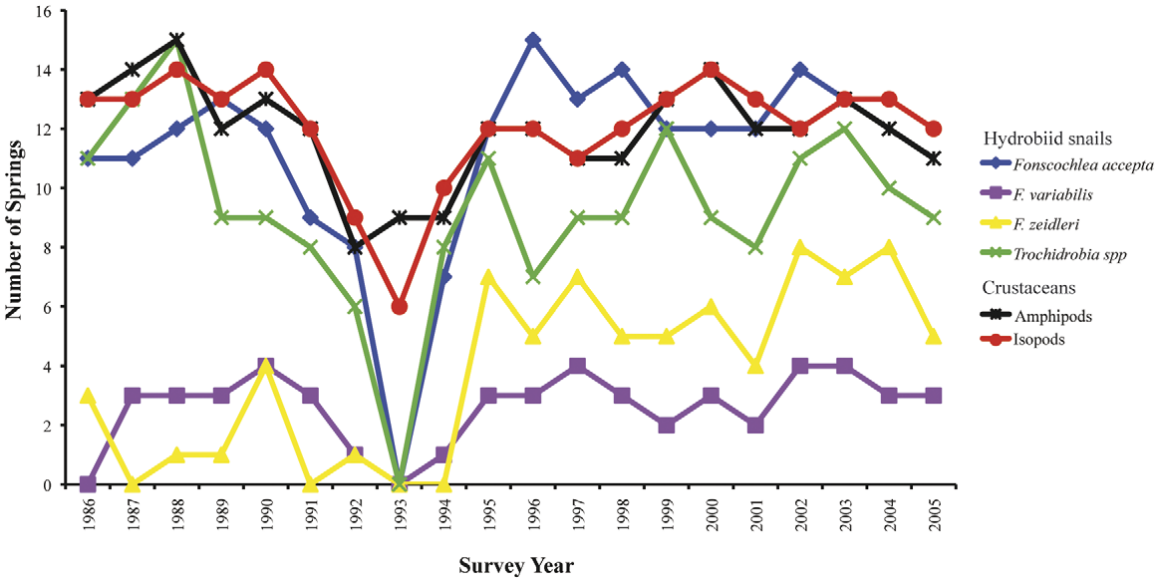
Survey data of the aquatic invertebrates endemic to our study springs. The hydrobiid snail fauna are identified to species (*Fonscochlea* sp) and genus (*Trochidrobia*), the endemic crustaceans are identified by type (amphipods and isopods). The data indicates whether or not the species were detected as being either present or absent in the springs.

Consistent with regional arid Australia, flooding in our study area is periodic with a major flood occurring approximately every 10–25 years and significant local flooding every 8–10 years [40]. In late 1992 (October–December) the region received almost 3.5 times the median annual rainfall (long term average = 126 mm, 1992 = 425 mm) [41], which resulted in large scale flooding throughout the Hermit Hill spring complex and surrounds in December 1992. The long term faunal monitoring program of the springs' endemic invertebrates revealed a catastrophic decrease in the occurrence of all hydrobiid snail species from the Hermit Hill springs in 1993, the year immediately following the flood (see Fig. 3). Recovery of hydrobiid populations following the flood (i.e. presence in springs) of all but one species was observed to begin by 1994 (Fig. 3). The impact of the December 1992 flood was not limited to just the hydrobiid fauna with declines also observed in the endemic crustacean species (amphipods and isopods, Fig. 3).

Our genetic sampling incorporates years both pre (1988–1990) and post (1995, 2002–2006) this devastating flood event. Sampling gaps exist immediately prior to (1991) and following (1994) the 1992 flood and post flood sampling was most intense 10 years after the flood. Apart from 1988–1990 and 1995, no other historical genetic samples exist for these springs. Despite these gaps, the first year sampled after the flood (i.e. 1995) enables post flood consequences to be clearly defined.

### DNA extraction and microsatellite genotyping

Total genomic DNA was extracted from homogenates of entire snails (approx. 3 mm diameter) using either DNeasy Tissue Kits (QIAGEN) or NucleoSpin Tissue Kits (Macherey-Nagel). All DNA samples were screened with a panel of nine variable microsatellite markers described in Worthington Wilmer *et al.* [42] and using protocols described in Worthington Wilmer *et al.* [39]. Samples were run on a GE Healthcare MegaBACE™ 4000 automated capillary sequencer and visualised and scored using MegaBACE Fragment Profiler v 1.2 software (GE Healthcare).

Due to the condition of snails collected from 1988–1990 and 1995 and potentially degraded DNA, all individuals from these years (and a subset of modern samples) were genotyped twice independently to ensure consistency of amplification and scoring. Loci and/or individuals for which there were amplification inconsistencies or scoring problems were excluded from the final data set.

### Statistical analyses

Prior to general analysis the data set was checked for genotyping and typographic errors using micro-checker [43]. Observed and expected heterozygosity and deviations from Hardy-Weinberg equilibrium (HWE) were calculated using GenePop on the Web (http://genepop.curtain.edu.au) [44]. The observed number of alleles in a sample is highly dependent on sample size. To account for differences in snail numbers between years in our study springs, within year genetic diversity was measured as an estimate of allelic richness, which calculates the number of alleles independent of sample size following the rarefaction procedure of El-Mousadik & Petit [45] (implemented in FSTAT v2.9.3, [46]). Inbreeding coefficients (F_IS_ values) for each spring's temporal samples were also calculated and their significance assessed using 10,000 permutations in FSTAT v2.9.3 [46].

### Temporal genetic differentiation within each spring

To test whether within spring genetic diversity estimates changed as a result of the flood devastation and subsequent recovery, temporal data for each spring was split into either pre (1988–1990) or post (1995, 2002–2006) flood groupings and significance of differences in allelic richness, observed heterozygosity and F_IS_ values were assessed. Before any pre or post flood group comparisons could be made, it was important to determine if data/years could be legitimately pooled into the pre and post flood categories. Therefore, significance of each of these genetic diversity variables was initially tested among years within each temporal (pre- and post flood) grouping for each spring using a Tukey's test conducted in R v 2.13.1 [47]. If no significant differences were found between years within each temporal grouping then significant differences between the pre and post flood groups for each spring were assessed using permutation exact tests (as they provide strong control for Type 1 error rates) with 10,000 permutations (implemented in FSTAT v2.9.3 [46]).

Temporal genetic structuring among years within each of the focal springs was examined in three ways. First, pairwise exact tests of allelic composition among years were conducted using GenePop on the Web (http://genepop.curtain.edu.au) [44]. Second, population differentiation between years was quantified using F_ST_ as estimated by theta [48] and implemented in FSTAT v2.9.3 [46]. Significance of the overall F_ST_ value and pairwise comparisons was assessed using 10,000 permutations. The critical significance threshold for the pairwise values was maintained at 5% following correction for multiple simultaneous tests using the false discovery rate (FDR) control [49] implemented in the program Pairwise Multiple Tests v 1.3 [50]. We chose the FDR control over the more commonly used sequential Bonferroni correction as it allows for increased power across a large number of repeated tests [51].

Finally, temporal population clustering was estimated using the Bayesian assignment program STRUCTURE v2.2.2 [52], [53]. For each focal spring, we conducted three independent runs of STRUCTURE for values of *K* between 1 and 8 (the exact number for each spring depended on the number of years for which it had temporal samples) with a burn-in value of 100,000 and 200,000 further iterations. Based on the results from previous studies that found no difference between admixture and no-admixture [38], we used a no-admixture model. Variance values were compared across the runs to test for convergence. We estimated the most likely number of clusters by ranking the standardized mean of the log transformed posterior probability for each *K* [52].

### Estimating temporal effective population sizes

There are multiple methods available to infer effective population sizes (N_e_) from genetic data. Following Aspi *et al.* [54] we used four different programs, which utilise several different statistical methods of estimating N_e_ in the pre and post flood years for each of the focal springs. The programs were as follows 1) N_E_ESTIMATOR v1.3 [55] which provides a moment-based estimate of N_e_ [56]; 2) MCLEEPS v1.1 [57] which provides a maximum-likelihood estimate of N_e_. We used this program to compute the likelihoods for N_e_ values between 5 and 1000 in increments of 5 using 10,000 Monte Carlo replicates for each value of N_e_; 3) MNe v1.0 [58], [59] provides a pseudo-likelihood (point) estimate of N_e_. We set max N_e_ to 10,000; 4) TVMP [60] which provides a Bayesian coalescent-based estimate of N_e_. We assumed a model of constant population size and used 10,000 MCMC replicates with 50 output updates. Standard errors on all N_e_ estimates were calculated in Excel.

Low levels of marker (allelic) diversity associated with a small number of loci may compromise the precision and accuracy of N_e_ estimates for these programs (see review [61]). However, we are looking for consistent differences in N_e_ between pre and post flood populations not the values of N_e_ *per se*.

### Spatial genetic differentiation within each year

In order to examine the population dynamics of the snails pre and post flood, spatial genetic structuring among the focal springs within each of the sample years was also investigated. If extensive mixing occurred due the flood, then pre flood population structure is predicted to be stronger than that post flood. If founder events are important in post flood population dynamics then we anticipate spatial structure will be stronger following the flood rather than before. We estimated spatial population structure by assessing the significance of pairwise F_ST_ values among springs within years using the same method described above for temporal estimates.

## Results

### Amplification success and exclusion of loci

All contemporary post flood snails (2002–2006) were successfully amplified and scored at the nine loci. Independent repeat amplifications of a subset of contemporary samples revealed no amplification inconsistencies or allelic drop out. In the pre flood (1988–1990) and 1995 snails four out of the nine loci either failed to amplify successfully (all Hex labelled primers) or produced inconsistent results across independent runs and were excluded from the analysis. For consistent pre and post flood group data comparisons, we excluded these 4 loci from the 2002–2006 data sets. All subsequent analyses and results presented therefore pertain to the reduced (5) loci datasets. However, summary genetic diversity statistics (allelic richness, observed and expected heterozygosity and inbreeding coefficients) for the contemporary samples across all 9 loci are provided (Supporting Information Table S1).

### Genetic diversity estimates

#### Individual springs all years

Estimates of allelic richness within individual springs and years ranged from 1.9 (West Finniss 026, 1990)–3.7 (Sulphuric 012, 2003), observed heterozygosities ranged from 0.180 (Bopeechee 004, 2006)–0.336 (Dead Boy 005, 1990) and F_IS_ values ranged from −0.612 (West Finniss 026, 1990) to 0.311 (Sulphuric 012, 1988/89) (Table 1). There was no evidence for linkage between any of the pairs of loci. Significant deviations from HWE were detected in one locus in 2 years in Bopeechee 004, Dead Boy 005 and West Finniss 026 and at one locus in a single year in Sulphuric 012 and 026 with there being no consistent pattern as to either the locus or sampling year in which the deviations were found. Detailed anatomical studies have shown that *F. accepta* is a dioecious species [27] and taken with the HWE results supports an outcrossing breeding system.

#### Pre and Post Flood Group Comparisons

Results of the Tukey's tests showed that there were no significant differences for any of the three genetic diversity estimates between years within either the pre and post flood categories for any of the five springs. Therefore testing for significant differences between the pre and post flood groups for each spring could continue. Permutation testing of genetic diversity statistics showed highly significant differences in pre and post flood estimates of both allelic richness and inbreeding coefficients in four of the five focal springs (Table 2). In those four springs mean allelic richness was higher in post flood samples. Similarly, inbreeding coefficients were consistently closer to zero in post flood years. Overall these results suggest a trend of increasing genetic diversity and a potentially more stable random mating structure in snail populations following the flood event. No significant differences in heterozygosity estimates in pre and post flood years were observed in any of the springs. The lack of any definitive change in heterozygosity even though there has been a change in allelic diversity is not unexpected. Heterozygosity is not as sensitive to the sampling effects caused by the loss or gain of rare alleles [12] and has been shown to be resistant to change by either single or multiple founder events (see [62] and references therein).

**Table 2:** Mean genetic diversity and inbreeding coefficients (F_IS_) between pre and post flood snail populations.

	Pre Flood	Post Flood	P value
Bopeechee 004			
Allelic Richness	2.4	3.1	**0.01**
Het Obs	0.475	0.426	0.85
F_IS_	−0.161	0.002	**0.03**
Dead Boy 005			
Allelic Richness	2.5	2.4	0.26
Het Obs	0.508	0.395	0.09
F_IS_	−0.118	0.01	0.15
Sulphuric 012			
Allelic Richness	2.1	2.8	**0.01**
Het Obs	0.568	0.563	0.64
F_IS_	−0.4	−0.016	**0.02**
Sulphuric 024			
Allelic Richness	2.6	3.1	**0.01**
Het Obs	0.577	0.464	0.053
F_IS_	−0.287	−0.048	**0.02**
West Finniss 026			
Allelic Richness	2.4	3.2	**0.02**
Het Obs	0.536	0.451	0.21
F_IS_	−0.192	0.017	**0.049**

Significant values highlighted in bold.

Het Obs = observed heterozygosity.

### Temporal genetic differentiation

Pairwise exact tests of allele frequencies among years within springs showed highly significant differences among all pre flood years in all springs where a temporal series was available (Table 3). Highly significant differences were also found among all but two pre and post flood year pairwise comparisons (Table 3). The only non-significant comparisons were between the years 1988 v 2005 and 2006 in the spring West Finniss 026. Furthermore, no significant differences in allele frequencies were recorded among any of the post flood years in any spring with the exception of one year (2003) for West Finniss 026.

**Table 3:** Exact tests of microsatellite allele frequencies. P values across 5 loci.

Bopeechee 004	Pre Flood			Post Flood				
	1988	1989	1990	2002	2003	2004	2005	2006
1989	**0.000**							
1990	**0.000**	**0.000**						
2002	**0.000**	**0.000**	**0.000**					
2003	**0.000**	**0.000**	**0.000**	0.569				
2004	**0.000**	**0.000**	**0.000**	0.388	0.405			
2005	**0.000**	**0.000**	**0.000**	0.942	0.092	0.230		
2006	**0.000**	**0.000**	**0.000**	0.282	0.073	0.559	0.179	

Dead Boy 005	Pre Flood			Post Flood					
	1988	1989	1990	1995	2002	2003	2004	2005	2006
1989	**0.000**								
1990	**0.000**	**0.000**							
1995	**0.000**	**0.000**	**0.000**						
2002	**0.000**	**0.000**	**0.000**	0.054					
2003	**0.000**	**0.000**	**0.000**	0.189	0.385				
2004	**0.000**	**0.000**	**0.000**	0.199	0.758	0.888			
2005	**0.000**	**0.000**	**0.000**	0.124	0.710	0.866	0.871		
2006	**0.000**	**0.000**	**0.000**	0.527	0.073	0.111	0.383	0.355	

Sulphuric 012	Pre Flood	Post Flood					
	1988/1989	1995	2002	2003	2004	2005	2006
1995	**0.002**						
2002	**0.000**	0.073					
2003	**0.000**	0.103	0.542				
2004	**0.000**	0.243	0.218	0.101			
2005	**0.000**	0.257	0.292	0.063	0.230		
2006	**0.000**	0.204	0.956	0.164	0.239	0.529	

Sulphuric 024	Pre Flood			Post Flood				
	1988	1989	1990	2002	2003	2004	2005	2006
1989	**0.000**							
1990	**0.000**	**0.000**						
2002	**0.000**	**0.000**	**0.000**					
2003	**0.000**	**0.000**	**0.000**	0.459				
2004	**0.000**	**0.000**	**0.000**	0.919	0.912			
2005	**0.000**	**0.000**	**0.000**	0.273	0.330	0.876		
2006	**0.000**	**0.000**	**0.000**	0.748	0.082	0.547	0.184	

West Finniss 026	Pre Flood			Post Flood			
	1988	1989	1990	2002	2003	2005	2006
1989	**0.000**						
1990	**0.000**	**0.000**					
2002	**0.028**	**0.000**	**0.000**				
2003	**0.000**	**0.000**	**0.000**	**0.042**			
2005	0.115	**0.000**	**0.000**	0.912	**0.002**		
2006	0.330	**0.000**	**0.000**	0.476	**0.034**	0.889	

Values in bold were significant (P<0.05) following correction using FDR control.

Pairwise estimates of F_ST_ values showed a near identical pattern to the allele frequency comparisons with highly significant differences in population structure recorded among all pre flood years and between all but one pre and post flood comparison in all springs (Table 4). In all springs, levels of differentiation were at least an order of magnitude higher in pre flood years (mean F_ST_ range 0.059–0.173) than post flood years (mean F_ST_ range 0.004–0.011) though four springs did record 1–2 significant post flood pairwise comparisons. The highest values were consistently between comparisons pre and post the flood (range 0.076–0.275) in all springs.

**Table 4:** Pairwise estimates of population subdivision among years within springs.

Bopeechee 004	Pre Flood			Post Flood				
	1988	1989	1990	2002	2003	2004	2005	2006
1988		0.091	0.178	0.295	0.312	0.315	0.245	0.309
1989	**0.041**		0.179	0.272	0.263	0.282	0.220	0.259
1990	**0.002**	**0.002**		0.281	0.274	0.286	0.254	0.258
2002	**0.002**	**0.002**	**0.002**		−0.005	0.007	−0.010	0.015
2003	**0.002**	**0.002**	**0.002**	0.493		−0.000	−0.001	0.008
2004	**0.002**	**0.002**	**0.002**	0.280	0.238		0.006	0.001
2005	**0.002**	**0.002**	**0.002**	0.863	0.071	0.159		0.014
2006	**0.002**	**0.002**	**0.002**	0.159	**0.030**	0.413	0.166	

Dead Boy 005	Pre Flood			Post Flood					
	1988	1989	1990	1995	2002	2003	2004	2005	2006
1988		0.041	0.058	0.220	0.232	0.226	0.220	0.225	0.205
1989	**0.001**		0.078	0.299	0.290	0.276	0.272	0.277	0.270
1990	**0.003**	**0.011**		0.187	0.219	0.204	0.207	0.217	0.179
1995	**0.000**	**0.000**	**0.000**		0.023	0.027	0.016	0.033	−0.006
2002	**0.000**	**0.000**	**0.000**	**0.022**		−0.006	−0.014	−0.004	0.007
2003	**0.000**	**0.000**	**0.000**	0.099	0.333		−0.010	−0.012	0.007
2004	**0.000**	**0.000**	**0.000**	0.114	0.526	0.820		−0.006	0.001
2005	**0.000**	**0.000**	**0.000**	**0.036**	0.674	0.580	0.673		0.003
2006	**0.000**	**0.000**	**0.000**	0.174	0.097	0.133	0.315	0.353	

Sulphuric 012	Pre Flood	Post Flood					
	1988/1989	1995	2002	2003	2004	2005	2006
1988/1989		0.057	0.088	0.076	0.070	0.072	0.091
1995	**0.003**		0.026	0.037	0.010	−0.001	0.014
2002	**0.000**	0.103		−0.003	0.004	0.003	−0.013
2003	**0.000**	0.092	0.409		0.015	0.017	0.007
2004	**0.000**	0.341	0.120	**0.047**		0.004	0.000
2005	**0.000**	0.467	0.316	**0.035**	0.214		−0.001
2006	**0.000**	0.197	0.872	0.069	0.131	0.504	

Sulphuric 024	Pre Flood			Post Flood				
	1988	1989	1990	2002	2003	2004	2005	2006
1988		0.077	0.157	0.214	0.170	0.176	0.174	0.259
1989	**0.000**		0.245	0.292	0.257	0.280	0.292	0.329
1990	**0.000**	**0.000**		0.122	0.137	0.133	0.128	0.176
2002	**0.000**	**0.000**	**0.000**		0.002	−0.002	0.010	−0.008
2003	**0.000**	**0.000**	**0.000**	0.235		−0.007	0.005	0.021
2004	**0.000**	**0.000**	**0.000**	0.812	0.859		−0.011	0.013
2005	**0.000**	**0.000**	**0.000**	0.248	0.278	0.833		0.031
2006	**0.000**	**0.000**	**0.000**	0.679	0.067	0.499	0.190	

West Finniss 026	Pre Flood			Post Flood			
	1988	1989	1990	2002	2003	2005	2006
1988		0.099	0.208	−0.006	0.022	−0.003	−0.011
1989	**0.031**		0.213	0.133	0.148	0.125	0.119
1990	**0.002**	**0.014**		0.197	0.251	0.214	0.259
2002	**0.024**	**0.010**	**0.002**		0.018	−0.007	0.001
2003	**0.010**	**0.010**	**0.002**	0.071		0.032	0.032
2005	**0.010**	**0.005**	**0.002**	0.869	**0.002**		−0.009
2006	0.150	**0.010**	**0.002**	0.540	0.055	0.793	

F_ST_ values across all loci on the upper matrix.

P-values for pairwise comparisons across all loci on the lower matrix.

Values in bold were significant (P<0.05) following correction using FDR control.

**Figure 4:**
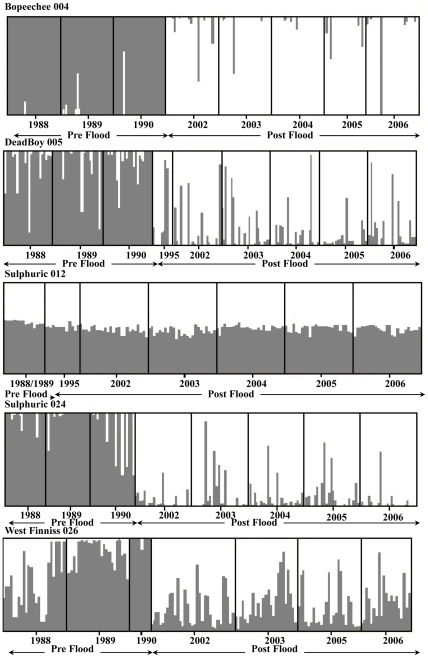
Results of the temporal assignment tests. Temporal assignment of individuals to *K* = 2 inferred clusters from the STRUCTURE analysis across all historical and contemporary snail samples within each of the five study springs. Grey indicates pre flood years (1988–1990) and white post flood years (1995, 2002–2006).

These results indicate substantial changes in the allelic composition and genetic structure in the snails in the years preceding the flood and in particular between pre and post flood populations. Results of the temporal assignment tests further support this scenario with individuals sampled in the years 1988–1990 assigned with an average probability of ≥90% to a pre flood cluster in four of the five focal springs (Figure 4, Supporting Information Table S2). Similarly individuals sampled in years 1995, 2002–2006 are assigned with an equally high probability (ave≥86%) to a contemporary post flood cluster in those same four springs. In three of these four springs the greatest increase in the posterior probability of the number of genetic clusters was found for *K* = 2 (Supporting Information Table S2). There are two notable exceptions to this pattern. Individual assignment tests for the spring Sulphuric 012 showed that all snails, regardless of sampling year could be being assigned to either a pre or post flood cluster with equal probability indicating (and supported by the estimates of *K*) a single non-differentiated population (Figure 4, Supporting Information Table S2). This is despite, however, the highly significant differences in allele frequencies and population structure that was found for this spring between the pre flood sample (1988/1989) and the contemporary post flood years using traditional frequentist estimates. This result for Sulphuric 012 most likely reflects the poor performance of assignment tests when F_ST_ values are low albeit significant (<0.1 between pre and post flood years, Table 4), samples size of the pre flood group is small and there are few loci involved [63]. The other exception was for the year 1988 in West Finniss 026, which can be assigned equally to either the pre and post flood clusters. As mentioned above this year is not significantly different in allelic frequencies or genetically differentiated from the last two contemporary sampling years (2005, 2006) and may indicate a return of this spring's *F. accepta* population to its earliest pre flood genetic composition.

### Temporal effective population sizes

**Figure 5:**
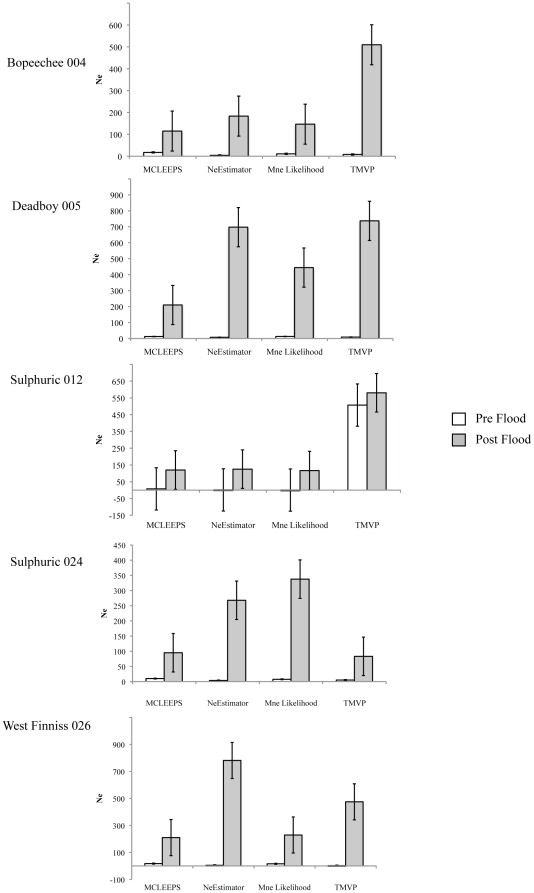
Comparison of temporal effective population size estimates within each of the five study springs. Values were estimated using four different statistical methods implemented in four different programs (see methods). White bars indicate values of N_e_ for pre flood (1988–1990) snail populations and grey bars indicate values of N_e_ for post flood (1995, 2002–2006) snail populations. Standard errors are indicated on each estimate.

As anticipated due to the low level of allelic diversity for our markers, each of the four programs gave widely varied estimates of effective population size between pre and post flood samples within each of the springs. However, regardless of the actual estimates the pattern for all programs across four of the five springs was identical; effective population sizes were found to be consistently larger after the flood than they were prior (Figure 5). The exception to this was spring Sulphuric 012 where while the actual values also mirror this pattern, the standard errors for each pre and post flood values overlap for all estimates. This is likely due to the small sample size of the pre flood group (n = 15) for this spring.

### Spatial genetic differentiation

Pairwise estimates of F_ST_ values among springs within years showed very different patterns of spatial structuring among pre and post flood populations (Supporting Information Table S3). In the pre flood years, evidence for spatial structuring among the springs varies from year to year. In 1988 only two significant differences in pairwise F_ST_ values among the springs were found, in 1989 significant F_ST_ values were found among all but two pairwise comparisons and in 1990 no significant differences among populations were recorded. For post flood years, however, the pattern was completely consistent. Highly significant differences in F_ST_ values were found among all springs in all years (1995, 2002–2006). The strong genetic structure recorded among springs in the post flood years, especially in 1995, provides evidence that founder events rather than mixing are more important in determining population dynamic processes of the snails following the flood.

## Discussion

This study has produced an intriguing and unexpected result. Our data clearly demonstrate changes in the levels of genetic diversity, allelic composition, genetic structure (both temporal and spatial) and effective population sizes of *F. accepta* populations within the same artesian springs. These changes may have been precipitated by the catastrophic flood that swept through these springs in 1992. This assertion is supported by the fact that snails from two springs sampled in 1995 clearly define genetically the post flood group and that genetic signature remains in populations sampled 10 years after this catastrophic disturbance. Contrary to predictions and expectations, our data shows that nearly all post flood snail populations had significantly higher allelic richness, inbreeding co-efficients that were closer to zero, little or no temporal changes in population structure and greater effective population sizes than their pre flood counterparts. Remarkably, our results are consistent with the possibility that a catastrophic disturbance, which resulted in a severe population crash, may have lead to enhanced levels of within population genetic diversity within the recovered populations. To our knowledge, this is the first result of its kind.

An additional finding was that the system may not have been in equilibrium prior to the flood. Data from the pre flood years showed annual changes in spatial structure among springs, increasingly negative inbreeding co-efficients and consistent significant differences in temporal allele frequencies and genetic structure. While the survey results (Fig. 3) appear to indicate that the invertebrate fauna was already in decline prior to the flood, a regression analysis (data not shown) found that only the *Trochidrobia* snail species exhibit a significant decline in the number of springs occupied during the 1989–1992 time period. While an earlier flood event was known to have occurred in the region in 1984 [40], the limitations of the historical sampling and the lack of available information regarding the conditions at our study site during that time prevents testing of processes influential in shaping snail population dynamics prior to the flood.

### Catastrophes pave the way to increased genetic diversity?

The previously undocumented increases in genetic diversity following the catastrophic flood can be challenging to explain mechanistically. The intuitive explanation is to assume that the flood acts as a mass dispersal or mixing event for these obligate aquatic organisms. Rather, floods are extreme mortality events. In addition to our survey data (Fig. 3), the detrimental impact of floods on the spring fauna is further supported by evidence obtained from a small sampling program run during an earlier flood (1984) event in the same region, which found complete mortality of all snails below the high water line [40]. According to Ponder and Hershler [40], the inundated Hermit Hill springs had been effectively “swept clean” of their invertebrate fauna. Finally, our analysis of spatial population structure clearly demonstrates that the flood does not facilitate genetic mixing. We therefore propose the combined effects of a massive decline in population size leading to a large amount of open spring habitat, followed by a shift in the effectiveness of alternative dispersal mechanisms combined with rapid population growth as the most likely explanation for the pattern we have observed.

Our previous spatial genetic studies showed dispersal by *F. accepta* was found to operate via two different mechanisms at two entirely different geographic scales [39]. Local dispersal (≤300 m) occurs via snails actively moving between springs that share direct aquatic connections. Long distance dispersal events (≥3 km) also occur in the system and are most likely facilitated by animal vectors (phoresy) [39]. During “normal” years most dispersal occurs among very large, standing populations in locally connected springs. For example, population densities of *F. accepta* have been recorded in outflows of two of the study springs (Bopeechee 004 and Dead Boy 005) of being up to 5.6±2.8 per cm^2^ (56,000/m^2^) [64]. Long distance dispersal events are detectable in the system during these years but migrant individuals make little impact on the genetic composition of the large established populations [39].

During flood years populations in inundated springs are decimated. As the floodwaters recede there remains a large amount of unoccupied spring habitat into which survivors can expand and recolonise. Evidence from the surveys shows that these populations rebounded quickly after the flood; by 1994 - 7/16 monitored springs had densities of *F. accepta* high enough to be detected again, by 1995 this had increased to 12/16 springs (Fig. 3, the above density figures were recorded in 1995). Immediately following the devastation, local dispersal among connected springs is likely to be non-existent due to affected populations being all but wiped out. However, long distance dispersal from less impacted or even undisturbed springs continues and brings migrant individuals carrying new alleles (following the “migrant-pool” model of recolonisation [65], [66]), which contribute to the genetic composition of the rapidly recovering populations. Given the design of the historical sampling, we are unable to identify the possible source springs for migrants for the recovering populations. Support for a multiple source argument comes from the assignment tests conducted for our more comprehensive contemporary spatial studies. This data showed that for 19 identified long distance migrants 15 different springs from throughout the Hermit Hill spring complex served as source populations [39]. The positive influence on genetic diversity of migrants originating from multiple source populations has been shown in a number of diverse species and systems including dogwhelk (*Nucella lapillus*, [67]), invasive populations of brown anoles (*Anolis sagrei*, [68]), lesser kestrels (*Falco naumanni*, [69]) and purple martins (*Progne subis*, [70]).

### Resilience versus resistance of artesian spring taxa

As with many biological communities [5], [6] different taxa within the artesian spring ecosystem display differential responses to catastrophic natural disturbances. The impact of the 1992 flood was not just limited to the hydrobiid fauna with declines also observed in the endemic crustacean species (amphipods and isopods, Fig. 3). Clearly, large scale flooding is devastating for the hydrobiid snails inhabiting the springs at the time, but they show rapid recovery (resilience). The crustacean species appear far more resistant to widespread flooding, disappearing from only half the monitored springs but taking 3–6 years to return to pre flood spring occupancy levels (Fig. 3). A study of anoles and spiders in the Bahamas found that the rate of recovery and recolonisation following a catastrophic hurricane was strongly proportional to the organism's dispersal ability [21]. This also appears to be true of the artesian spring taxa. The dispersal capacity of snails (as described above) underpins their rapid post flood recovery but also explains the lack of diversity and phylogeographic structure in *F. accepta* using lower resolution markers such as allozymes [28] and mitochondrial CO1 sequences [71], in contrast to the strong spatial structure detected using higher resolution microsatellites. Considerably less is currently known about the dispersal abilities of the endemic amphipods and isopods. However, a recent comparative study of the aquatic invertebrate fauna inhabiting the same springs, showed that in contrast to *F. accepta* there is significantly greater genetic divergence and phylogeographic structuring in both amphipod and isopod COI sequences [71]. This may be taken as indirect evidence for greater population persistence combined with poorer dispersal ability. Hence, while the susceptibility of the crustacean species to flood devastation may be less, their recovery is slower.

### Conclusion

Catastrophic disturbances can have an enormous variety of causes and effects. Consequently, ecosystems and species demonstrate an equally wide array of responses [72]. Our data suggests that the population genetic consequences for individual species to catastrophes may not be as predictable as we might have otherwise anticipated. In fact the response of individual species can be highly idiosyncratic and is likely to be influenced by both the ecology of species (e.g. whether they are resilient or resistant to disturbance) and the stability of the environment (i.e. frequency and intensity of disturbance regimes) in which they have evolved. The endemic fauna of the artesian spring ecosystem in arid Australia have evolved in an environment characterised by irregular but recurrent intense disturbances. For species such as *F. accepta* and possibly the other hydrobiid snails, catastrophic disturbances may instigate what might be described as a “population genetic reset”, with these seemingly devastating events ultimately acting as agents for the genetic diversification of populations.

## Supporting Information

Table S1 Genetic diversity estimates and inbreeding co-efficients for contemporary snails samples across 9 loci.
(XLS)

Table S2 Posterior probabilities and variances for models assuming various numbers of clusters (K) for each of the five study springs over all years. Statistics for each value of K represent average values over three separate runs. Assignment test inferred pre and post flood clusters represent average values over three separate runs where K = 2.
(XLS)

Table S3 Pairwise estimates of F_ST_ among springs within years.
(XLS)
